# On the Treatment of Certain Fractures

**Published:** 1910-06-25

**Authors:** W. H. Clayton-Greene


### Hospital Clinics.

### ON THE TREATMENT OF CERTAIN FRACTURES.

By W. H. CLAYTON-GREENE, M.B., F.R.C.S.

(A Lecture delivered at the Polyclinic on June 1, 1910.)

The branch of surgery with which I am dealing this afternoon is one rather calculated to put us out of conceit with ourselves. In most cases, indeed, the results are satisfactory, but in others, in spite of every care, we are unsuccessful, and it is chiefly to the type of cases in which those disappointing results occur that I wish to direct your attention. There are certain cases of fracture which are generally regarded as merely common fracture and are not submitted to any special operative treatment, but nevertheless, from the point of view of after-effects, are a great deal more serious than is generally recognised. If the average practitioner of experience were asked in what fracture he regarded operative treatment as most necessary he might reply that the fractures of the patella and of the olecranon were among the first. And perhaps to a certain extent he would be right, for the amount of strain upon the fibrous union is considerable, and there is a tendency for great separation of the fragments. But in cases of fracture of the olecranon in which operation is commonly recommended I have seen massage and movements give quite satisfactory results. On the other hand, there are fractures in which operative treatment is not enjoined, although as a matter of fact these are fractures which oftener require operative treatment even than the patella.

### The Question of Operation.

In judging whether operation is necessary in a given case, we are guided largely by the disability which will result if the case is left merely to manipulations and splints. Other factors, however, have to be taken into account, including the patient's age and the nature and demands of his occupation. In the case of the extremities it is not the question of shortening that is of the greatest importance, although that, of course, is a consideration, but the deviation from the normally straight lines. In fractures of the tibia angulation is of great importance, and even more important in fractures involving the bones of the forearm. A common deformity, for instance, seen in fractures upon the radius and the ulna is a sagging of the arm towards the ulnar side. This was mentioned and emphasised in the account of fractures written by Mr. Stanley Boyd in Treves's "Surgery"; but, so far as I know, that is the only text-book in which emphasis is laid upon this exceedingly common deformity. If the bones are allowed to unite, even with a moderate angulation, it will seriously interfere with the degree of pronation and supination permitted afterwards. This particular deformity is even more serious than actual overlapping of the bones, although, of course, if this overlapping implicates both the radius and the ulna it is much more serious because there is vicious union.

Let us first consider the question of fracture or injury in the region of the shoulder joint. This is very common. Within three or four months we may have half-a-dozen such cases. The patients are often somewhat stout, rendering the manipulation of the fragments, both for observation and treatment, a matter of difficulty. In these cases there is commonly a certain bruising and swelling. I emphasise the importance of the bruising because, in a simple dislocation, it is a rare thing to get any marked extravasation of blood. The feature is noteworthy, and I have seen several cases of fracture possessing it in which the diagnosis has been missed and treatment given for dislocation.

### Fractures of the Humerus.

The fractures which result from such injury as I have mentioned may be divided into three—fracture of the surgical neck, fracture of the anatomical neck of the humerus, and fracture of the neck of the scapula. The last is the least common. In the fracture of the surgical neck we have usually little difficulty in making a diagnosis; the chief difficulty arises in those cases in which there is fracture of the anatomical neck or of the edge of the glenoid cavity. In both these latter conditions there is flattening of the shoulder, the capsule of the joint will be torn, and—for the moment I am considering the fracture of the anatomical neck—a large, irregular portion of the head will be displaced by the rent in the capsule of the internal coracoid process. At first sight, therefore, one is inclined to diagnose the condition as a dislocation, and, this being done, attempts are made to reduce it. Unquestionably there is some result from the manipulations, and something seems to slip into place; usually it is the shaft of the bone slipping back into position. Of course, there is only one certain method of eliminating the question of fracture, and that is by submitting the patient to the *x*-rays. Personally, I will never now undertake a case of this kind unless I am allowed a free hand in the matter of radiography. With the great amount of swelling present and with the bones buried as they are in swollen tissues, it is difficult to diagnose by manipulation and quite impossible to treat. That is the first point I wish to emphasise. I have seen several of these cases diagnosed and treated as dislocations, and it is only when, after a period of six or seven weeks, the patient has not improved that the *x*-rays have been employed and have revealed the true state of affairs, which is invariably a dislocation of the head of the bone and an indifferently formed false joint. Such a condition is usually intolerable to the patients. In very few instances do they get any return of movement under those circumstances, and many of them suffer from severe brachial neuritis.

We may divide fractures of this order into three groups. In the first place we have the ordinary form of fracture of the surgical neck. In the second form, in addition to the fracture, the upper fragment is very markedly abducted. In the third form the head of the bone is displaced. We are indebted largely to Treves for recognising these three distinct types of fracture of the upper end of the humerus. I am not discriminating between fractures of the surgical and of the anatomical neck at the moment, and you will see my reason presently. If in any one of these fractures we have the two fractured surfaces lying in fair apposition, no special line of treatment is necessary. In some of these cases there may not even have been complete separation, and some of the remnants of the periosteum may be found acting as a temporary splint. In such cases we may get a perfect functional result, and never under any circumstances do they require operative interference.

In the second type of fracture we find that the upper fragment is markedly abducted. Any attempt to treat it upon the lines we adopted in the first instance—a splint applied to the outer side of the arm or a pad in the axilla—must necessarily result in malunion, because we now have two fractured surfaces fairly widely separated. These cases must be treated in abduction, and sometimes the arm is put above the patient's head—a position advocated by some American surgeons. As an alternative we have to consider the advisability of operation. By means of an operation, after abducting the arm to a considerable extent, and by fixing the fragments in position with a plate, we can then treat as in the first variety. I do not insist on the necessity for an operation if the patient is willing to submit to an exceedingly irksome position and have his arm considerably raised.

### The Third Type.

With regard to the third type of fracture, no manipulative proceeding, so far as I know, is here of the slightest value. It is difficult enough sometimes to effect reduction of the dislocation even when a free incision has been made, but I believe personally that it is absolutely impossible for us to effect a reduction of the dislocated head without operation. It has been stated that by manipulation the head of bone can be forced into position, but it is probable that in the cases thus described the head of the bone was never properly out of the capsule of the shoulder joint. One line of treatment which has been urged consists in first putting up the arm, allowing the two fractured surfaces to consolidate, and then, after seven or eight weeks have elapsed, attempting reduction by manipulative methods. From my own experience of many cases I believe that no reduction has ever been effected in an old dislocation of this kind, and that any attempt to reduce the displacement by manipulation would result in re-fracture. I said something to the same effect when I lectured on this subject a short time ago; and since then, on looking through the "Annals of Surgery," I came across an article stating that all the recorded cases in which this treatment had been adopted were fruitless, from the point of view of cure. In all the cases which had been examined the bone was found still to be out of position, and in two cases the actual condition of re-fracture had occurred. Manipulation, then, is out of the question if we are to arrive at a satisfactory result.

What are the alternatives? If the patient is left in this state he may get a species of false joint, a very imperfect substitute owing to the irregular outline of the fractured surfaces. And, moreover, he will have a piece of bone of considerable size abnormally situated in his anatomy. The degree of his future suffering depends largely upon the position which this fractured fragment assumes. If it is displaced well up beneath the coracoid process, he is certain to suffer from neuritic trouble. The operation consists of making a free incision, then manipulating the fragments into position, and finally fixing them by wires, screws, or plates. That is an ideal line of treatment, very nicely expressed in diagrams on paper, but when we actually operate on these cases we frequently find that there is comminution of the fragments to such an extent as to make it almost impossible to restore the normal configuration of the upper end of the humerus. In my own practice I have always had the fear that if I did make a mosaic of this puzzle of bone I should only be building up such a mass of callus formation that in the end the shoulder joint would become ankylosed. For that reason I have never adopted such a proceeding. In those cases where it is desired to preserve the fractured end of the bone I recommend the use of the traction hook which was invented by MacBurney, inserting it into the bone, and so reducing the dislocated head without damaging it. As these accidents often occur in subjects who are very ready to develop adhesions and get fixation of their joints after any injury, the best line of treatment is freely to excise this displaced end, and, having removed it, to smooth off the fractured end of the humerus, as one does in the operation for excision of the shoulder joint, and allow the wound to heal. A species of false joint is then formed between this broken end and the glenoid fossa, but the movement is impaired usually to so slight an extent that the man, if a workman, is able in most instances to earn his living. Generally speaking, he has no pain or discomfort to speak of, and the worst thing that happens to him is some muscular weakness due to associated damage of the circumflex nerve.

### Femoral Fractures.

The above illustrates one variety of fracture which demands some form of operative treatment. As a contrast to that and in conjunction with it, I will deal for a few moments with fractures of the upper end of the femur. Of these there are two varieties—the intra-capsular and the extra-capsular. The extra-capsular form—to take it first—results from the application of much violence to the great trochanter. I have known one or two cases which were due to the kick of a horse. Owing to the force outside and to the resistance of the acetabulum within, the neck is driven into the trochanter like a wedge, and the great trochanter is split so that the upper part is often completely detached. It is stated in the descriptions of this form of fracture that the limb may be either internally or externally rotated, according to the position it was in at the time of the injury. But while this may be so, it must be remembered that the limb is nearly always in the position of adduction in those cases in which there is impaction. When speaking of fractures of the upper end of the humerus, I said that they were exceedingly liable to be overlooked, and the same thing applies to the fractures of the upper end of the femur. There may be considerable bruising and pain, yet we may be unable to detect any crepitus and perhaps unable to find any shortening. If we measure the two limbs, one in abduction and the other in adduction, we shall find a disparity under ordinary circumstances, and if we are dealing with an adducted limb in a case of fracture a shortening of an inch or half an inch is quite easily overlooked. I know of one patient with a bruised hip who was treated for sciatica, and it was not until an *x*-ray photograph was taken that the true state of affairs was revealed. Indeed, I have seen a sufficient number of these cases to put me on my guard as to injuries of the hip. And I refuse to offer any opinion as to the nature of the damage until an *x*-ray photograph has been taken.

### Treatment.

Having eliminated the danger of overlooking the real character of the injury, the next question is the possible line of treatment. Ordinarily we are told to put the patient to bed and use a weight extension. This, however, does not take into account the fact that many of these cases are impacted and in a position of adduction. In some of the text-books we are told that an impaction should be left alone. In the majority of cases this would be well, perhaps, if impaction were all, but we have to consider the deformity of adduction also, and I have noticed that in many of these patients after the injury has healed—and it does not heal very well—there is a constant limp and they are unable to abduct the limb freely. As a result we have traumatic coxa vara, and I do not regard a splint or doubly inclined plane as satisfactory in themselves. These fractures, as I have said, do not heal very well. You may have seen a number of such cases in which there has been considerable functional disability. In many instances this is due to excessive callus formation. It is still a debateable point as to how far that callus formation is the result of passive movement. There are two distinct schools of thought with regard to this matter, one stating that passive movement should be always practised in the treatment of fractures, and the other that passive movement has been practised to excess and should not be employed quite so vigorously as has been advocated. I am not in want of instances to prove that callus formation does follow this movement. I have known certain injuries to the wrist-joint, for example, in which the carpal bones have been broken, and which have been treated for sprain by passive movement, show extensive callus formation at the scaphoid. Passive movement has a distinct field of application, but these fractures are not always amenable to it, and in no case do I advocate passive movement until a fortnight or three weeks after the receipt of the injury. A line of treatment adopted by certain of the Continental schools in extra-capsular fractures, and one which I favour, is first to anæsthetise the patient, disimpact the fracture, and then to fix him up with the limb in almost full abduction.

### Paracephalic Fracture.

Intra-capsular fracture, in the majority of cases, occurs in elderly people. But it is met with also in childhood, and occasionally in the young and apparently healthy adult. In the child it is a variety of separation of the upper femoral epiphysis, and it leads to the most marked form of traumatic coxa vara. But, as you are aware, this is a fracture which does not unite as a general rule, possibly owing to the age of the patient in the majority of cases. By reason of this fact it is a fracture in which it is not wise to practise immobilisation. In elderly people we consider the general condition rather than the fracture, but, of course, we are not justified in doing this in the case of the young subject. In his case it becomes our object to try and obtain bony union between these fractured surfaces. In the case of the child we put on a weight extension and keep him in that position for a month, the limb being slightly abducted. But in the case of the adult this line of treatment is very rarely sufficient. It is, however, difficult to draw a line and say that in a particular case we are to try and get bony union and in another not. On seeing a number of these elderly people—and by elderly I mean people of fifty and upwards—suffering from the after-effects of intra-capsular fracture, we cannot but be very dissatisfied with their condition. We recently had in a man of fifty-six, who was treated on the ordinary approved lines for the elderly subject, and he is now permanently crippled, with an enormous mass of callus formation, and a limb which is quite useless as a means of progression. It is questionable whether, even in a man of fifty-six, we are justified in sacrificing him to permanent crippledom because we are afraid to face the risk of an operation. The fact must be kept in mind that the great thing to aim at in such cases is the fixing of the fragments so that they will not slip or move. An operation is often performed by which the neck of the bone is exposed from the front, and the screw run through from that point to the head. But this inflicts further damage upon the already damaged tissues. On the other hand, the great trochanter is very accessible, being the most superficial part of the femur in this region. It is easily arrived at from the outside, and we merely require to take into account the nature of the junction of the femoral neck with the base of the great trochanter to enable us to channel through the bone in that situation, and introduce a nail, which is generally employed, or a long screw. With such a mode of procedure we need only take into consideration the obliquity and the direction of the femoral neck. Of course, one would naturally take a radiograph and make sure that there was no natural peculiarity in the conformation of the bone. Before the operation is done it will be necessary to put on a weight extension in order to drag the femur down to its proper position. I dare say it occurs to you that it will be a difficult matter to hit off exactly the distance from the base of the great trochanter to the head. But that need not concern us in the least. I believe the most successful cases have been those in which the nail penetrated through the head and actually became impacted in the acetabulum.

One Continental surgeon, indeed, advocates that the nail should always be driven so far home that it becomes fixed to the acetabulum, and says that this proceeding interferes with the movement to a much smaller extent than might have been expected. Without going so far as that, I think it wiser certainly to err on the side of pushing the nail too far than of pushing it not far enough. With the nail well pushed in we have got a very strong internal splint—sometimes two in the case of a strong man—uniting the fractured surfaces together so that it is almost impossible for them to come apart. No amount of movement afterwards can affect them. It must be clear to you, I think, that, accepting the necessary risks of any operative interference, this is a line of treatment which is very well worth practising in a large number of cases. It is far more preferable, at all events, than to allow the patient to become a helpless cripple owing to the fact that we have been afraid to introduce a nail obliquely into one of the bones of the body.

